# Design and Control of an Adaptive Knee Joint Exoskeleton Mechanism with Buffering Function

**DOI:** 10.3390/s21248390

**Published:** 2021-12-15

**Authors:** Yapeng Wang, Wei Zhang, Di Shi, Yunhai Geng

**Affiliations:** 1 Research Center of Satellite Technology, Harbin Institute of Technology, Harbin 150080, China; gengyh@hit.edu.cn; 2 Beijing Xinfeng Aerospace Equipment Co., Ltd., Beijing 100854, China; 3 School of Mechanical Engineering and Automation, Beihang University, Beijing 100191, China; BY1507114@buaa.edu.cn (W.Z.); shidi@buaa.edu.cn (D.S.)

**Keywords:** knee exoskeleton, adaptive mechanism, joint center compensation

## Abstract

A knee exoskeleton with an adaptive instantaneous rotation center and impact absorption is used for rehabilitation. Due to the human knee joint’s special physiological structure and motion characteristics, the exoskeleton mechanism needs to be designed for both static and dynamic aspects. Therefore, a novel knee exoskeleton mechanism was designed. To adapt to the rotation center of the knee joint, a mechanism with cross-configuration was designed according to the equivalent degree of freedom and the stiffness of the springs was calculated by its combination with gait motion, so that the average force of the human body was minimized. A dynamic model of the exoskeleton was established. To overcome the uncertainty in the parameters of the human and robotic limbs, an adaptive controller was designed and a Lyapunov stability analysis was conducted to verify the system. A simulation was conducted and experimental results show that the tracking error of the knee joint angle between the actual and desired trajectory was within the range of −1 to 1 degree and indicate the effectiveness of the controller.

## 1. Introduction

The human knee joint bears the force that is transferred between the thigh and lower leg, which is very important for human gait. An advanced knee exoskeleton helps those who suffer from intentional trauma, stroke, spinal cord injury, etc. to return to normal gait and life [1,2,3]. The research on the physiological characteristics of the knee joint [4,5,6,7] point out two key problems for the design of a reasonable knee exoskeleton. Firstly, the instantaneous rotation center of the knee joint changes with the rotation of the joint in the sagittal plane, leading to a mismatch between the exoskeleton and the human body. Secondly, the impact force of the knee joint when walking is large, which affects the strength of the exoskeleton and causes discomfort for the person.

Due to the change of the instantaneous center, structures with multi-links and redundant degrees of freedom are mainly adopted. For example, Dong Hai Wang et al. [6] and Pina Martinez et al. [8] designed a mechanism by cam-fitting and implemented the mechanism through actual anatomical samples; Dong Jin Hyun et al. [9] and Ramanpreet Singh [10] directly fitted the instantaneous center trajectory of the knee joint through four-bar linkages; Matthew Eschbach et al. [11] and Jiun-Yih Kuan et al. [12] compensated for the movement of the instantaneous center’s trajectory through four connecting links and a slider. However, due to the use of a rigid body, subject to the difference of physiological structure, fitting range, and accuracy, there will be a certain mismatch between the fitting range and the applicability that is greatly affected by individual. In addition, the structures with redundant degrees of freedom are adopted, such as the RoboKnee designed by Jerry E. Pratt et al. [13], the quasi-passive knee joint robot developed by Aaron M. Dollar et al. [14], and the Schmidt coupling adopted by M. Yalcin in Turkey [15]. However, when the weight of the lower part of the knee–ankle joint mechanism is large, the lower leg will bear more force when walking.

To compensate for the knee’s joint rotation and tibia axial impact force, the series elastic actuator (SEA) is widely used in rotary impact research and the non-metallic elastic parts are used for the axial impact. The elastic part of the elastic actuator, which imitates the knee joint, has the function of reducing the rotational vibration when landing and preventing a possible increase in metabolic cost, user pain, and discomfort. The RoboKnee adopts a linear drive series elastic actuator with a realizing rotary buffer, LOPES [16] adopts a cable-driven series elastic actuator, and Nikos Karavas of Pisa University in Italy [17] designed a swing-type spring compression mechanism, which has low impedance characteristics and high drive bandwidth and can adapt well to impact force and have smaller impedance characteristics compared to the human body. Kamran Shamaei et al. [18] of Yale University calculated the elastic damping coefficients of the gait impact process and swing process based on experiments and then designed a switching process of the two elastic damping elements to realize quasi-passive gait motion. However, the linear drive and pull-rope-driven series elastic actuator is larger, while the swing spring type has nonlinear stiffness. Many studies [19,20,21,22,23,24] adopt compact and nearly linear elastic parts and most of them adopt an in-plane curve design, but most of the design methods for an elastic part currently involve simulation and experiment. There are two types of curve bending, including continuous bending and repeated bending. The tibia axial load buffering of the knee joint is carried out by tibia cartilage and meniscus, in which the meniscus plays a greater role. In [25], the ability of the meniscus to cushion impact and eliminate vibration was studied. Under the axial load of the tibia, the meniscus can bear up to 75% of the impact. In 2014, Chen Bing et al. [26] designed a foot robot with shock absorption and in 2016, Li Fei et al. [27] designed a prosthetic mechanism with shock absorption. The buffering function of the bionic meniscus and cartilage mechanism can reduce the impact of the mechanism and simultaneously increase the gait matching and comfort of the person appropriately.

This study proposes the design and control of an adaptive knee joint exoskeleton mechanism with a buffering function. To the best of the authors’ knowledge, the contributions of this article are summarized below.

(1) A cross-configuration structure was designed to adapt to the change of the instantaneous center, with the ability to be adapted for a wide range of people.
(2) A structure with a buffering function was designed to absorb impact when walking, which can bear the weight of the mechanism and reduce its complexity.
(3) An adaptive controller was designed to compensate for the uncertainty and external disturbances of the model.

The rest of this article is organized as follows. The physiological characteristics of the knee joint are presented in Section 2. Section 3 presents the design of the mechanism. The design of the adaptive controller is presented in Section 4. The simulation and experiments used to verify the proposed method are presented in Section 5. The conclusions are drawn in Section 6.

## 2. Physiological Characteristics of the Knee Joint

**Figure 1:**
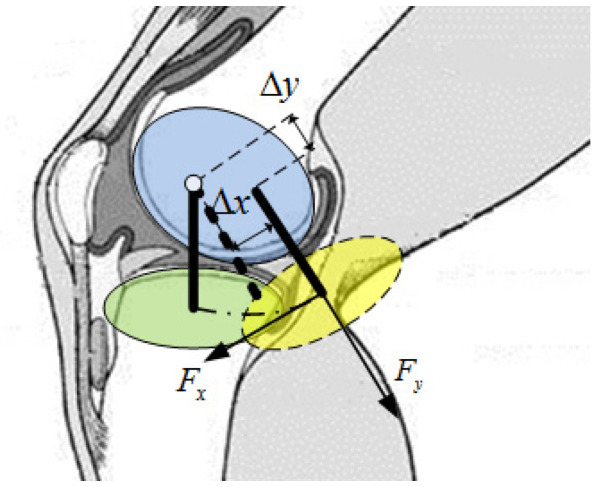
Translations of the lower leg.

The knee joint is a large and complex joint in the human body. It is mainly composed of the lower femur, upper tibia, meniscus, patella, cruciate ligament, joint capsule, and related muscles and cartilage [28]. The knee joint is a hinge joint, and the relative motion of the leg is a composite movement of rotation and translation due to the offset and radius change of the rotation center, as well as a small internal and external rotation. The main motion is rotation, and the knee offset is in two directions during gait [28]. The authors in [5] summarized the previous literature and concluded that the lower leg of the human body shifts about 15 mm in the plane perpendicular to the rotation axis with a change in the knee joint angle. Therefore, when a simple rotation axis is adopted for knee joint orthosis, the tibia directional force will be greater and the gait will cause the strap to become displaced. The motion angle of the knee joint is relatively large but may be set to 0~60°, considering the gait motion is small. The two-translational motion of the leg relative to the sagittal plane are Av and Ay, and the human–computer interaction force in the corresponding directions are Fx and Fy, which are shown in Figure 1.

When a normal person weighs 70 kg, the peak value of instantaneous torque during gait can reach more than 42 Nm. At this time, muscles and ligaments have the function of lubrication and buffering. In some patients with stroke, incomplete spinal cord injury, and other symptoms, the lack of strength in the knee joint easily leads to a movement imbalance. At the same time, the meniscus is composed of fibrocartilage, which can increase the depth of the articular fossa, stabilize the knee joint, cushion vibration, and lubricate the joint. With the help of surface curvature changes, the meniscus can greatly improve its shock- and vibration-buffering ability. Under the axial load of the tibia, the meniscus can bear up to 75% of an impact.

In this study, we need to measure the translation of the lower leg in two directions, the sagittal plane, and the kinematics and dynamics data of the gait process, which can be used for the design basis of the adaptive mechanism.

### 2.1. Leg Translation Measurement

**Figure 2:**
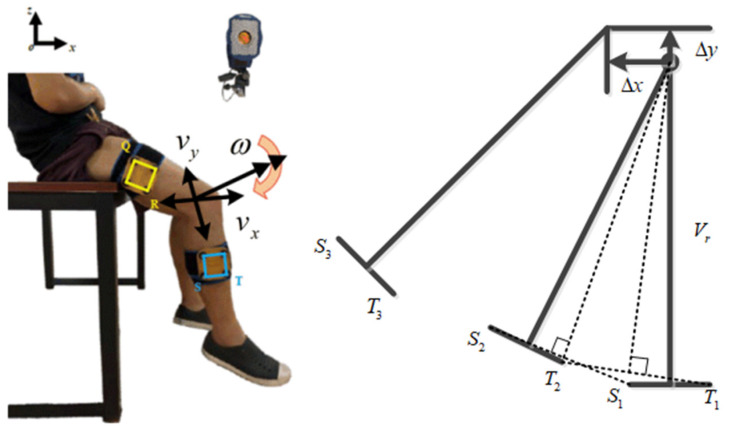
Measurement of leg translation.

Due to the fact that the object has three degrees of freedom in the plane, which includes one rotation and two translations, the essence of the change of the instantaneous center of the knee joint is caused by the translation of the leg during the joint rotation. Therefore, in this study, motion analysis was used to measure the translation change of the leg in the sagittal plane with rotation. There are four points in the thigh and four points in the lower leg. The final measurement of the relative motion of the S and T points relative to the Q and R points is shown in Figure 2. The flexion of the knee joint is defined as the positive direction. With an increase of 5 degrees in the knee joint angle, the translation distance of the lower leg relative to the initial position was measured in two directions of the sagittal plane. In this way, the process of measuring the movement within the range of 0 to 60° and the movement values measured along the X and Y directions are shown in Figure 2. The translation trajectory curves in the two directions can be obtained by cubic curve-fitting.

In this study, we further measured the hip and knee joint motion data under general walking speed by using motion analysis. The gait data of walking 3 km/h on a treadmill were recorded using the Helen Hayes lower limb sticking method. Taking the measured data as the starting point with the right foot off the ground, a total of 20 groups of data were obtained. There was a group of data that had a large deviation, so it was excluded. In this study, the average of the gait data was calculated and analyzed.

### 2.2. Impact Force Experiment and Stiffness Fitting

The influence of different walking speeds on the knee joint angle–torque was further measured. Since the human gait frequency is 95–125 steps per minute, the corresponding pace will also change [29]. Therefore, in this study, the knee joint motion data of three individuals were measured with 80, 90, and 100 steps per minute. The landing force of the knee joint under three gait frequencies was obtained by a motion capture system and dynamometer. The height and weight of the three persons is given in Table 1.

## 3. Design of the Knee Joint Mechanism

An instantaneous center adaptive cross mechanism with a buffer was designed. The structure’s design was divided into two parts: the design of the cross-configuration structure to adapt to the instantaneous center change of the knee joint and the vertical buffer elastic part of the cross-configuration to absorb the impact during walking.

### 3.1. Cross-Configuration Design

**Figure 3:**
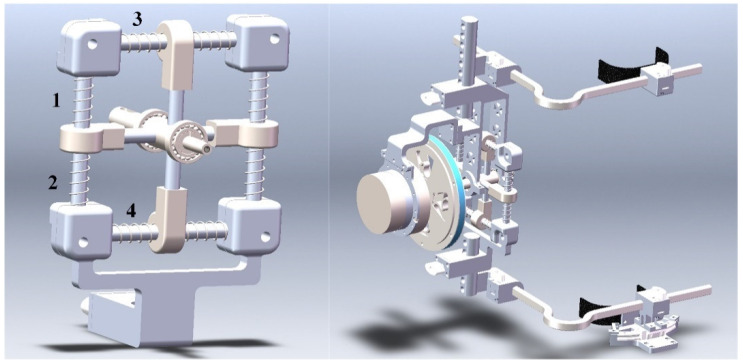
CAD model of the knee joint.

According to the physiological structure of the knee joint as described in Section 2.1, the instantaneous center change of the knee joint is equivalent to the translational motion of the leg in the sagittal plane. Therefore, to realize the tracking of translational leg motion for different people, the knee exoskeleton mechanism was designed with four degrees of freedom: two rotations and two translations. The two rotations were the main rotation of the knee joint in the sagittal plane and the internal and external rotation connected to the ankle. The two translation pairs were connected in series behind the sagittal rotation pair to form a cross shape to reduce the space being occupied. The increased redundant degrees of freedom were constrained by springs. Compared to the Schmidt coupling and four-bar linkage, this configuration could be more easily adapted for a wider range of people and could bear the weight of the lower part of the knee joint because of the reduced complexity of the mechanism. The cross-configuration is shown in Figure 3. No. 1 and No. 2 are elastic parts that run parallel to the lower leg, and No. 3 and No. 4 are elastic components that run perpendicular to the lower leg. When being worn, the centerline of the rotating pair will coincide with the bulge on the side of the lower end of the femur, as far as possible. The outer side of the rotating pair of the mechanism will be connected to the rotational elastic part, the reducer, and the motor in turn. Furthermore, combined with the gait characteristics and mechanism configuration, the mechanism will need to meet the minimum error force of the mechanism both when static and during gait. Therefore, the corresponding stiffness values for the four springs need to be calculated by dynamics.

**Figure 4:**
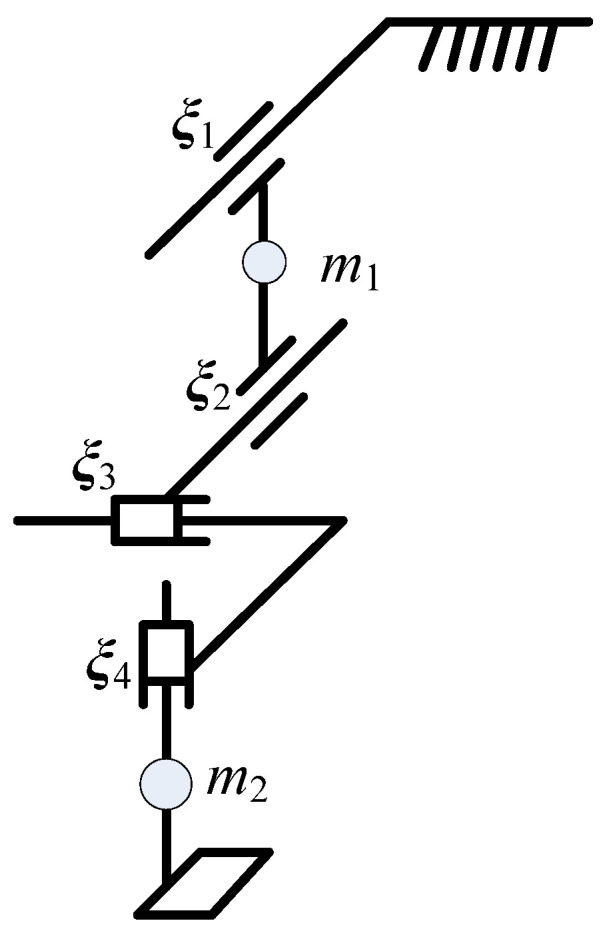
Simplified graph of the degrees of freedom in sagittal plane.

The exoskeleton mechanism was simplified, and only the degree of freedom in the sagittal plane was retained, as shown in Figure 4. In the quasi-static motion of the human knee joint, the amount of spring compression depended on the weight of the mechanism and the shape of the human knee joint. When the stiffness of the spring was small, inertial forces caused by gait and gravity would cause the human leg to feel the tension through the bandage. When the spring was moderate, the tension would not be felt at some angles. During the dynamic motion of the human knee joint, the amount of spring compression depended on the dynamic force and the shape of the human knee joint. When the dynamic force matched the shape of the human joint, the force felt by the lower leg would have a small range of variation. Considering that the mechanism will mainly be used in dynamic motion, this study was conducted according to dynamics and also tested the feeling of static force.

The cross-configuration provided two translational forces for gait, so the appropriate stiffness could be calculated to balance the gait inertia force, gravity, and knee joint deformation according to the human gait data. Firstly, the absolute velocity, angular velocity, and angular acceleration of the human knee joint were substituted into the Lagrange equation to calculate the force at the cross point. To calculate the force of two sliding motions in the cross mechanism, the motion parameters of the sliding motion were set as shown in Figure 4. $\theta_{1}$ and $\theta_{2}$ are the rotational motion parameters of the hip and knee joint. $\theta_{3}$ and $\theta_{4}$ are the horizontal slip motion parameters. $m_{1}$ and $m_{2}$ are the mass of the thigh and shank. $d_{1}$ is the distance from the hip rotation pair to the centroid. $l_{1}$ is the length of the first rod. $d_{2}$ is the distance from the knee joint down to the center mass of the shank. The parameters above were obtained through the CAD model. Let the screws of the four motion parameters be $\xi_{1}$ − $\xi_{4}$, respectively. Combined with the gait data, the four motion parameters could be used to calculate the driving force of each joint. The motion of the different screws under each center of mass in the object–coordinate system was calculated as Equation (1). There was only one joint in front of $m_{1}$ and four joints in front of $m_{2}$.

(1) $$\{\begin{array}{l} {\xi_{1}}^{\prime }=Ad_{e^{{\hat{\xi }}_{1}\theta_{1}}\cdot g_{1}}\cdot \xi_{1}\\ {\xi_{i}}^{\prime }=Ad_{e^{{\hat{\xi }}_{1}\theta_{1}}\cdot e^{{\hat{\xi }}_{2}\theta_{2}}\ldots e^{{\hat{\xi }}_{i}\theta_{i}}\cdot g_{2}}\cdot \xi_{i}i=1,2,3,4 \end{array}$$

where Ad() is an adjoint representing the operator.

The Jacobian matrix of each center of mass:

(2) $$\{\begin{array}{l} Jac_{1}=\left[{\xi_{1}}^{\prime }\right]\\ Jac_{2}=\left[{\xi_{1}}^{\prime }\;{\xi_{2}}^{\prime }\;{\xi_{3}}^{\prime }\;{\xi_{4}}^{\prime }\right] \end{array}$$

The calculation formula for the kinetic energy and potential energy is as follows:

(3) $$\{\begin{array}{l} T=0.5\cdot \left[Jac_{1}^{T}\cdot M_{1}\cdot Jac_{1}+Jac_{2}^{T}\cdot M_{2}\cdot Jac_{2}\right]\\ V=d_{1}\cdot \left(1-\cos\theta_{1}\right)\cdot m_{1}\cdot g+\left(l_{1}\cdot \left(1-\cos\theta_{1}\right)+d_{2}\cdot \left(1-\cos\left(\theta_{1}+\theta_{2}\right)\right)\right)\cdot m_{2}\cdot g \end{array}$$

It could be concluded that the forces in the two directions of the cross are

(4) $$\{\begin{array}{l} F_{3}=\frac{d}{dt}\cdot \left(\frac{\partial L}{\partial {\dot{\theta }}_{3}}\right)-\frac{\partial L}{\partial {\dot{\theta }}_{3}}\\ F_{4}=\frac{d}{dt}\cdot \left(\frac{\partial L}{\partial {\dot{\theta }}_{4}}\right)-\frac{\partial L}{\partial {\dot{\theta }}_{4}} \end{array}$$

The position of the third and fourth joints was zero, so the velocity and acceleration were zero. The difference between the inertia force, gravity force, and anti-clastic force was calculated in the forward and backward direction (*x*) and vertical direction (*y*), so that the difference was as close to zero as possible. When the ideal function value was zero, the spring compensates for the inertia force and gravity, and the human body feels the least tension. The calculation formula is as follows:

(5) $$F_{obj}={\left(F_{x}-k_{x}\cdot \Delta x\right)}^{2}+{\left(F_{y}-k_{y}\cdot \Delta y\right)}^{2}$$

where $F_{x}$ and $F_{y}$ are the forces when the springs in two directions are compressed, and x and y are the horizontal and vertical translation of the leg at the knee joint. In this case, the spring stiffness values in two directions could be obtained as follows:

(6) $$\{\begin{array}{l} k_{x}=3N/\mathrm{mm}\\ k_{y}=4.7N/\mathrm{mm} \end{array}$$

### 3.2. Design of the Vertical Butler Part

**Figure 5:**
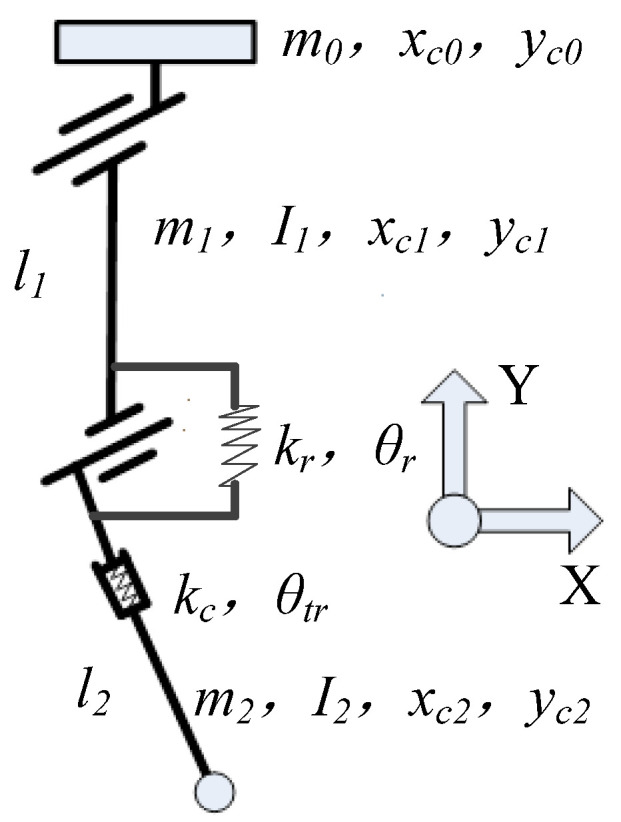
Simplified mechanism with buffer parts.

The vertical elastic buffer of the cross-configuration absorbed the impact of the rotating spring in the landing process. The mechanism was simplified, as shown in Figure 5. The parameters were as follows. Since the length of the participants’ thighs and legs were similar, there was only a slight difference in weight. Therefore, it could be said that the length of the two members were the same, i.e., $l_{1}=l_{2}=l$. The center of mass was in the middle of the links, and their coordination was $\left(x_{c0},y_{c0}\right)$, $\left(x_{c1},y_{c1}\right)$, and $\left(x_{c2},y_{c2}\right)$. The mass and inertia moment of the links are $m_{0}$, $m_{1}$, $I_{1}$, $m_{2}$, and $I_{2}$, respectively. At the same time, due to the small rotation angle, the spring with stiffness $k_{r}$ basically bears the weight of $m_{1}$ and $m_{2}$ vertically, so it was considered that all movements in the X direction were not affected by the spring movement.

According to the Lagrange calculation formula, the torque of the knee joint’s rotating elastic part $k_{r}$ and the force of the vertical elastic part during the buffering process were calculated. Firstly, the Lagrange dynamic equation is obtained as follows:

(7) $$\{\begin{array}{l} T=\frac{1}{2}m_{0}y_{0}{}^{2}+\frac{1}{2}m_{1}\left(x_{c1}{}^{2}+y_{c1}{}^{2}\right)+\frac{1}{2}m_{2}\left(x_{c2}{}^{2}+y_{c2}{}^{2}\right)+\frac{1}{2}I_{1}\theta_{1}{}^{2}+\frac{1}{2}I_{2}\theta_{2}{}^{2}\\ V=m_{0}gy_{0}+m_{1}gy_{c1}+m_{2}gy_{c2}+\frac{1}{2}k_{r}\theta_{r}{}^{2}+\frac{1}{2}k_{c}\theta_{c}{}^{2}\\ L=T-V \end{array}$$

where

(8) $$\begin{array}{l} x_{0}=0,y_{0}=2l\cos\frac{\theta_{r}}{2}+\theta_{c},\\ x_{c1}=\frac{l}{2}\sin\frac{\theta_{r}}{2},y_{c1}=\frac{3l}{2}\cos\frac{\theta_{r}}{2}+\theta_{c},\\ x_{c2}=\frac{l}{2}\sin\frac{\theta_{r}}{2},y_{c2}=\frac{l}{2}\cos\frac{\theta_{r}}{2} \end{array}$$

Furthermore, the force at $k_{r}$ is obtained as follows:

(9) $$\begin{array}{ll} -k_{r}\cdot \theta_{r} & =m_{0}{\ddot{y}}_{0}l\sin\frac{\theta_{r}}{2}+\frac{1}{2}m_{1}\left[\frac{l}{2}{\ddot{x}}_{c1}\cos\frac{\theta_{r}}{2}+\frac{3l}{2}y\sin\frac{\theta_{r}}{2}\right]\\ & +\frac{1}{2}m_{2}\left[\frac{l}{2}{\ddot{x}}_{c2}\cos\frac{\theta_{r}}{2}+\frac{l}{2}{\ddot{y}}_{c2}\sin\frac{\theta_{r}}{2}\right]\\ & +\frac{1}{4}I_{1}{\ddot{\theta }}_{1}+\frac{1}{4}I_{2}{\ddot{\theta }}_{2}+m_{0}g2l(-\sin\frac{\theta_{r}}{2})\\ & +m_{1}g\frac{3l}{4}(-\sin\frac{\theta_{r}}{2})+m_{2}g\frac{l}{4}(-\sin\frac{\theta_{r}}{2}) \end{array}$$

where

(10) $$\{\begin{array}{l} {\ddot{y}}_{0}=\frac{{\dot{\theta }}_{r}{}^{2}l}{2}\cos\frac{\theta_{r}}{2}+l\left({\ddot{\theta }}_{r}-{\ddot{\theta }}_{c}\right)\sin\frac{\theta_{r}}{2}\\ {\ddot{y}}_{c1}=\frac{3{\dot{\theta }}_{r}{}^{2}l}{8}\cos\frac{\theta_{r}}{2}+\frac{3{\ddot{\theta }}_{r}l}{4}\sin\frac{\theta_{r}}{2}-{\ddot{\theta }}_{c}\\ {\ddot{y}}_{c2}=\frac{{\dot{\theta }}_{r}{}^{2}l}{8}\cos\frac{\theta_{r}}{2}+\frac{{\ddot{\theta }}_{r}l}{4}\sin\frac{\theta_{r}}{2} \end{array}$$

Therefore, the equation could obtain the following relationship, where the greater the upward acceleration of the compression spring, the greater the rotation torque of the spring:

(11) $$k_{r}\cdot \theta_{r}\propto {\ddot{\theta }}_{c}$$

In addition, referring to the SEA mode in [30], $k_{c}$ could reduce the impact force, and the force decreased with a reduction in the spring stiffness in order to increase the movement stroke for a smaller force. The spring $k_{c}$ was compressed first after landing, and then acceleration $\ddot{\theta }$ was generated by the action of $m_{0}$ and $m_{1}$, which made the rotating spring force increase. Considering the simplified vertical spring $k_{r}$ acting on $m_{0}$ and $m_{1}$, the approximate values were as follows:

(12) $${\ddot{\theta }}_{c}\approx \frac{k_{c}\cdot \theta_{c}-c_{c}\cdot {\dot{\theta }}_{c}}{m_{0}+m_{1}}$$

According to the equation, there were four cases:

(13) $$\{\begin{array}{l} k_{c}\to \infty :{\ddot{\theta }}_{c}=0,{F_{r}}^{\prime }\approx F_{r}\\ k_{c}{>c}_{0}\cdot \left(m_{0}+m_{1}\right)g:{F_{r}}^{\prime }>F_{r}\\ k_{c}<c_{0}\cdot \left(m_{0}+m_{1}\right)g:{F_{r}}^{\prime }<F_{r}\\ k_{c}\to 0,c_{c}>0:{F_{r}}^{\prime }<F_{r} \end{array}$$

In the latter two cases, the spring stillness was too small to support the weight. The mechanism needed to adopt more stiffness and dumping in order to increase the vibration period, thus minimizing the influence on the spring with the stiffness $k_{r}$.

To calculate the stiffness, it was necessary to provide the boundary conditions of the buffer process. The boundary conditions included the initial landing height, $h_{off}$, of 0.04 m; the maximum compression distance of the meniscus, $h_{\max}$, was 3 mm, so the actual compression amount of spring was not greater than this value; the compression time in the landing process was 0.17 s for the 3 km/h gait data, so the actual compression time of the vertical spring should not have been exceeded. As the spring stiffness was increased with the buffer force, the spring stiffness should be selected within the boundary conditions.

The stiffness of the compression spring was calculated under boundary conditions. From the initial landing height, it was assumed that the kinetic energy and potential energy of $m_{0}$ and $m_{1}$ above the buffer of the compression spring were as follows. If the cushion rotating spring was ignored, the calculation result would be more desirable.

(14) $$k_{c}\geq 2\left(m_{0}+m_{1}\right)\cdot g\cdot \frac{h_{\max}+h_{off}}{h_{\max}{}^{2}}=5.13\times 10^{5}$$

The amplitude *A* of the compression vibration could be calculated as follows. Combining *A* and $h_{\max}$, the compression time of 0.01 s could be calculated, which was far less than 0.17 s.

(15) $$A=\Delta x\left(k_{c}\right)-\frac{\left(m_{0}+m_{0}\right)\cdot g}{k_{c}}=\frac{\sqrt{{\left(m_{0}+m_{0}\right)}^{2}\cdot g^{2}+2k_{c}\cdot \left(m_{0}+m_{0}\right)\cdot g\cdot h_{off}}}{k_{c}}$$

Since nitrile butadiene rubber (NBR) is widely used as a damping buffer, this material was used in this study. The damping effect of NBR is 0.15 times that of critical damping. 

## 4. Adaptive Controller Design

The knee exoskeleton was actuated with a single degree of freedom (DOF) and the cross-configuration structure was passive, which made it difficult to control. Therefore, the dynamics for the simplified 1 DOF mechanism were established and an adaptive controller was designed to compensate for the uncertainty and external disturbances of the model.

### 4.1. State Space Model

**Figure 6:**
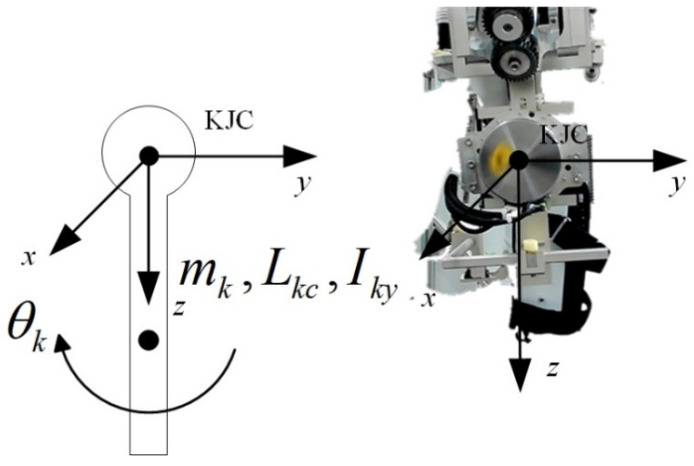
Mechanical structure and prototype of the knee exoskeleton.

A coordinate system was established at the center of the knee joint (KJC) (Figure 6), with the sagittal axis as y and the vertical axis as z, and the knee exoskeleton was simplified to rotate around the sagittal axis. The mass of the rotating part is $m_{k}$, the distance between the center of mass and KJC is $L_{kc}$, and the moment of inertia is $I_{ky}$.

The kinetic energy is

(16) $$T_{k}=\frac{1}{2}\left(m_{k}L_{kc}^{2}+I_{ky}\right){\dot{\theta }}_{k}^{2}$$

The potential energy is

(17) $$U_{k}=-m_{k}gL_{kc}\cos\theta_{k}$$

The dynamical equation is

(18) $$M_{r}{\ddot{q}}_{r}+C_{r}{\dot{q}}_{r}+G_{r}=T$$

where $q_{r}=\theta_{k}$, and

(19) $$M_{r}=m_{k}L_{kc}^{2}+I_{ky},C_{r}=0,G_{r}=gL_{kc}\sin\theta_{k}$$

$q_{d}$ is the desired angle for $q_{r}$. All signals were bounded. The generalized variables could be defined as $x_{1}=q_{r}$ and $x_{2}={\dot{q}}_{r}$ for practical applications.

(20) $$\{\begin{array}{l} {\dot{x}}_{1}=x_{2}\\ {\dot{x}}_{2}=M_{r}^{-1}\left(u-C_{r}x_{2}-G_{r}\right) \end{array}$$

### 4.2. Adaptive Controller Design

Step 1: The error was defined as $\;z_{1}=x_{1}-x_{1d}\;$, and the first Lyapunov function was defined as

(21) $$V_{1}=\frac{1}{2}z_{1}^{2}$$

The virtual variable was designed as

(22) $${\bar{x}}_{2}=-c_{1}z_{1}+{\dot{x}}_{1d}$$

Here, $c_{1}\in \mathbb{R}$. 

To avoid an explosion of terms [31], ${\bar{x}}_{2}$ was passed through a first-order filter as

(23) $$\tau_{2}{\dot{x}}_{2d}+x_{2d}={\bar{x}}_{2},x_{2d}\left(0\right)={\bar{x}}_{2}\left(0\right)$$

Here, $\tau_{2}\in \mathbb{R}$.

Step 2: After defining $z_{2}=x_{2}-x_{2d}$, the second Lyapunov function was defined as

(24) $$V_{2}=\frac{1}{2}M_{r}z_{2}^{2}$$

Using the property of the dynamic equation [32], a calculation was determined.

(25) $${\dot{V}}_{2}=M_{r}z_{2}{\dot{z}}_{2}+\frac{1}{2}{\dot{M}}_{r}z_{2}^{2}=z_{2}\left(u-C_{r}x_{2d}-G_{r}-M_{r}{\dot{x}}_{2d}\right)$$

The control law was

(26) $$u=-c_{2}z_{2}+M_{r}{\dot{x}}_{2d}+C_{r}x_{2d}+G_{r}$$

Here, $c_{2}\in \mathbb{R}$.

It was impossible to obtain an accurate dynamics model. Thus, the control law was

(27) $$u=-c_{2}z_{2}+{\hat{M}}_{r}{\dot{x}}_{2d}+{\hat{C}}_{r}x_{2d}+{\hat{G}}_{r}+f$$

Here, $f={\tilde{M}}_{r}{\dot{x}}_{2d}+{\tilde{C}}_{r}x_{2d}+{\tilde{G}}_{r}$ was used to determine the error between the nominal and actual models. The radial basis function neural network was exploited to approximate *f.*

(28) $$\hat{f}={\hat{W}}_{k}\varphi_{2}\left(x_{1},x_{2},x_{1d},{\dot{x}}_{1d},x_{2d}\right)$$

The updating law was designed:

(29) $${\dot{\hat{W}}}_{k}=-\Gamma \left(\varphi z_{2}+\theta \hat{W}\right)$$

In the above, ${\hat{W}}_{k}$ was estimated by Equation (29).

## 5. Simulation and Experiment

### 5.1. Simulation and Results

**Figure 7:**
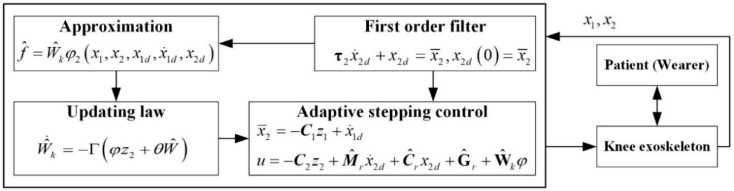
The control diagram.

The control parameters were chosen as $m_{k}=1.2871\;\mathrm{kg}$, $I_{ky}=1\;\mathrm{kg}\cdot m^{2}$, and $L_{kc}=0.503\;m$ to calculate the dynamics parameters $M_{r}$, $C_{r}$, and $G_{r}$ for the actual model, and the nominal model was chosen as ${\hat{M}}_{r}=0.3M_{r}$ and ${\hat{G}}_{r}=0.4G_{r}$. The updating law was chosen as Γ=0.01 and θ=0.01. The diagram of the control system is shown in Figure 7. The desired trajectory of the knee joint for normal gait was generated using the parametric method [33]. The walking speed was 1 kph and the gait cycle was 2.8 s. The mass of the shank and thigh were estimated using the inertial parameters of an adult human body.

**Figure 8:**
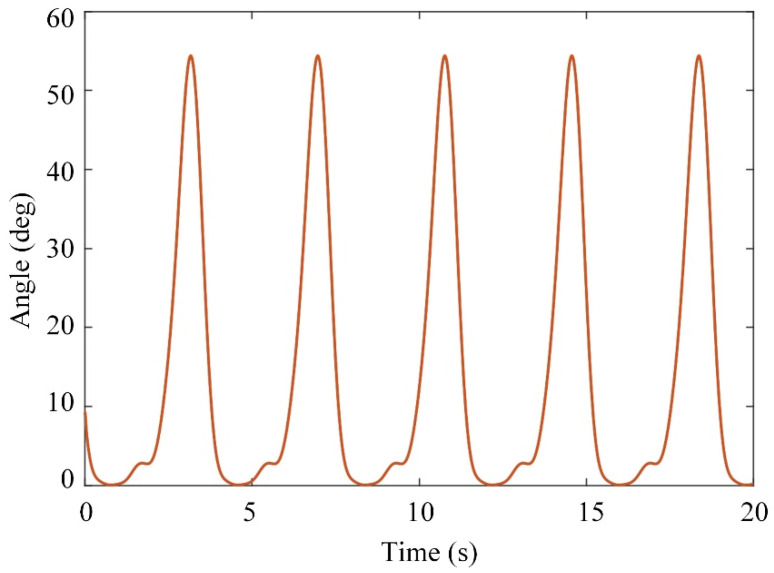
Desired trajectory.

**Figure 9:**
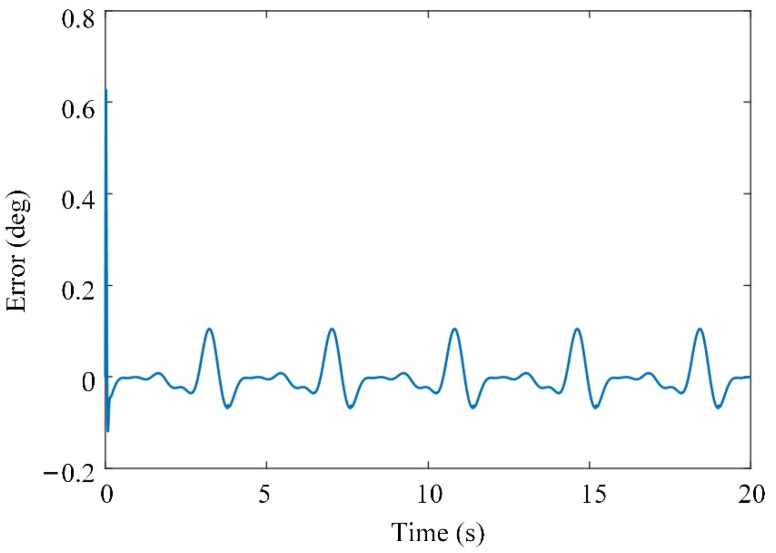
Tracking error.

**Figure 10:**
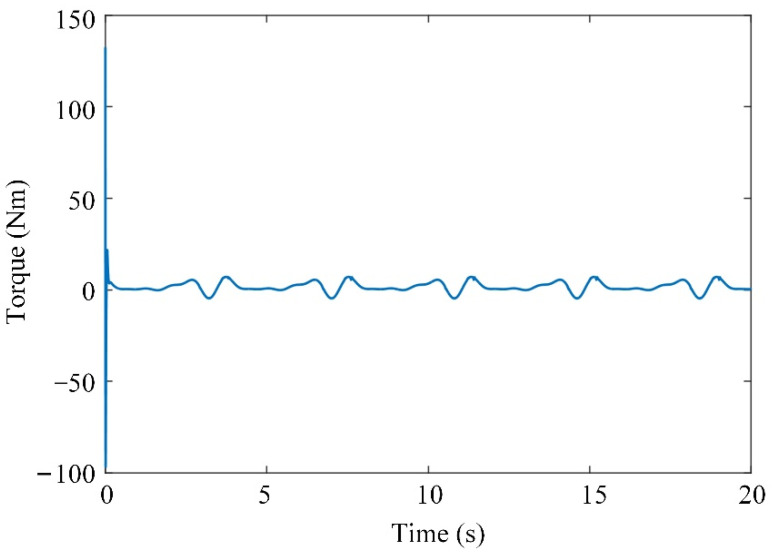
Generated torque.

The input trajectory, tracking error, and generated joint torque of the system are shown in Figure 8, Figure 9 and Figure 10, respectively. The initial tracking error exists. The simulation results show that the tracking error of the system can be controlled within a certain range by using the adaptive controller to compensate for the uncertainty of the model, and the system has uniformly bounded stability. The designed controller can realize the motion control of the knee joint exoskeleton.

### 5.2. Experimental Results

**Figure 11:**
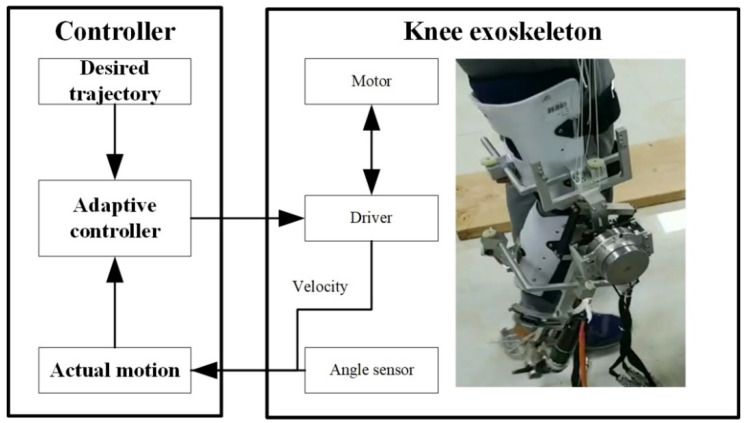
The prototype and control system.

**Figure 12:**
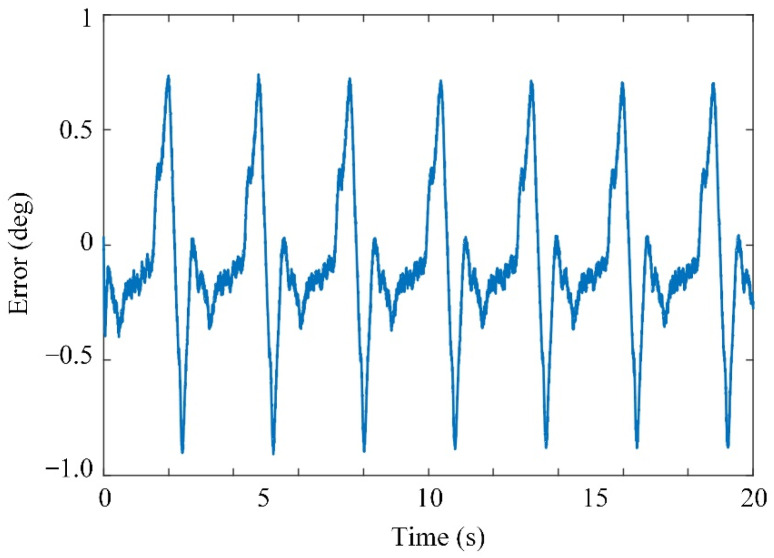
Tracking error.

**Figure 13:**
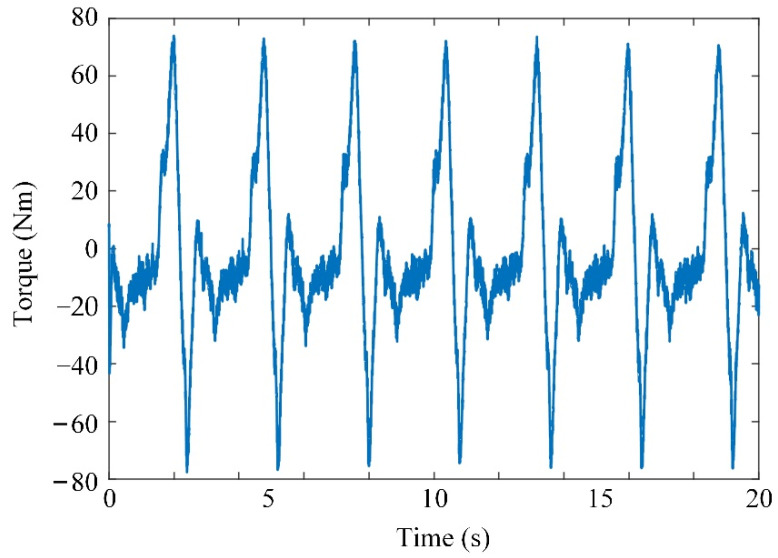
Generated torque.

The prototype and control system were designed for the experiments, as shown in Figure 11. The knee exoskeleton was driven by a rotational joint with a Maxon EC FLAT 90 motor. A joint angle sensor was used to measure the actual output angle of the exoskeleton. The angular velocity was measured by the encoder of the motor. A subject participated in the experiment by wearing the knee exoskeleton for the trajectory tracking. It was assumed that the subject had no knee control, so the knee joint of the subject was driven by the knee exoskeleton. The error between the desired and actual angles is shown in Figure 12 The generated torque is shown in Figure 13. The experimental results show that the tracking error of the knee joint angle between the actual and desired trajectory is within the range of −1 to 1 degree, and the system has uniformly bounded stability. The designed controller can realize the motion control of the knee exoskeleton. The torque in Figure 10 is much smaller than that in Figure 13 and the tracking error in Figure 9 is smaller than that in Figure 12. In the simulation, the mass of the shank and thigh of the subject was estimated, however, the parameters are not accrual, which makes the difference between the simulation and experiment valid. Meanwhile, the musculoskeletal system in humans is flexible, not rigid, so the disturbances exerted by the subject was time-varying and switched the torques to adapt to these variations.

### 5.3. Limitations

This study is not without limitations. Although the participant said he felt comfortable wearing the knee joint mechanism during the experiment, more subjects need to participate in the future in order to optimize the mechanism. The experimental results show a larger tracking error than the simulation. The control parameters that are chosen by the simulation need to be optimized in the future.

## 6. Conclusions

In this study, through analyzing the physiological structure and movement characteristics of the knee joint during human gait, an adaptive cross-configuration with redundant degrees of freedom was designed for the accompanying translational motion of the lower leg with rotation in the gait process with the a less complex mechanism, compared to 4-bar linkage or SEA. The dynamic model of the exoskeleton was established. To overcome the uncertainty in the parameters of the human and robotic limbs, an adaptive controller was designed and a Lyapunov stability analysis was conducted to verify the system. The simulation and experimental results indicate the effectiveness of the controller.

## Figures and Tables

**Table 1:** Height and weight of testers.

	Subject A	Subject B	Subject C
Height (cm)	175	183	185
Weight (kg)	75	87	70
